# HIV-1 anchor inhibitors and membrane fusion inhibitors target distinct but overlapping steps in virus entry

**DOI:** 10.1074/jbc.RA119.007360

**Published:** 2019-01-29

**Authors:** Dirk Eggink, Ilja Bontjer, Steven W. de Taeye, Johannes P. M. Langedijk, Ben Berkhout, Rogier W. Sanders

**Affiliations:** From the ‡ Laboratory of Experimental Virology, Department of Medical Microbiology, Amsterdam University Medical Centers (Amsterdam UMC), Location AMC, University of Amsterdam, Meibergdreef 9, 1105 AZ Amsterdam, The Netherlands,; the ¶ Department of Microbiology and Immunology, Weill Medical College of Cornell University, New York, New York 10065, and; § Pepscan Therapeutics BV, Zuidersluisweg 2, 8243 RC Lelystad, The Netherlands

**Keywords:** human immunodeficiency virus (HIV), drug resistance, inhibition mechanism, virus entry, fusion protein, membrane fusion, peptides, anchor inhibitor, antiviral drug, fusion peptide (FP)

## Abstract

HIV-1 entry into cells is mediated by the envelope glycoprotein (Env) and represents an attractive target for therapeutic intervention. Two drugs that inhibit HIV entry are approved for clinical use: the membrane fusion-inhibitor T20 (Fuzeon, enfuvirtide) and the C-C chemokine receptor type 5 (CCR5) blocker maraviroc (Selzentry). Another class of entry inhibitors supposedly target the fusion peptide (FP) and are termed anchor inhibitors. These include the VIRIP peptide and VIRIP derivatives such as VIR165, VIR353, and VIR576. Here, we investigated the mechanism of inhibition by VIR165. We show that substitutions within the FP modulate sensitivity to VIR165, consistent with the FP being the drug target. Our results also revealed that VIR165 acts during an intermediate post-CD4–binding entry step that is overlapping but not identical to the step inhibited by fusion inhibitors such as T20. We found that some but not all resistance mutations to heptad repeat 2 (HR2)-targeting fusion inhibitors can provide cross-resistance to VIR165. In contrast, resistance mutations in the HR1-binding site for the fusion inhibitors did not cause cross-resistance to VIR165. However, Env with mutations located outside this binding site and thought to affect fusion kinetics, exhibited decreased sensitivity to VIR165. Although we found a strong correlation between Env stability and resistance to HR2-based fusion inhibitors, such correlation was not observed for Env stability and VIR165 resistance. We conclude that VIRIP analogs target the FP during an intermediate, post-CD4–binding entry step that overlaps with but is distinct from the step(s) inhibited by HR2-based fusion inhibitors.

## Introduction

The HIV type 1 (HIV-1) is the causative agent of the acquired immunodeficiency syndrome (AIDS) and over 37 million people are currently infected worldwide. Although progress has been made in the development of treatment strategies, the capacity of the virus to become resistant to drugs is a major problem in the fight against AIDS (1). Drugs used in the current combination antiretroviral therapy target the viral integrase, protease, or reverse transcriptase. Because of the acquisition of resistance against these drugs and the possibility of cross-resistance to drugs directed against the same viral proteins, other drug targets need to be explored.

The envelope glycoprotein (Env)^3^ represents such an additional target. Env is a trimeric complex consisting of three gp41 subunits and three noncovalently attached gp120 subunits. Env mediates membrane fusion and virus entry, a process that is initiated by binding of gp120 to the CD4 receptor. This interaction induces a conformational change revealing the binding site for a chemokine co-receptor, generally CXCR4 or CCR5 (2, 3). Receptor-triggered conformational changes in gp41 involve two heptad repeat regions (HR1 and HR2) in gp41. First, a trimeric coiled-coil consisting of three HR1 domains is formed and the fusion peptide (FP) is inserted into the target cell membrane. Next, the three HR2 domains associate with the HR1 trimer core, resulting in the formation of a stable post-fusion six-helix bundle (4–7) that brings together the viral and cellular membranes and provides the free energy for membrane fusion (8).

Targeting the HIV-1 entry process has several advantages over other targets. First, in contrast to, for example protease inhibitors, the virus is blocked before the viral genome is integrated into the host cell genome, thus preventing the establishment of latent viral reservoirs. Second, entry inhibitors do not need to enter cells, unlike reverse transcriptase, integrase, and protease inhibitors. Third, the entry process comprises distinct steps that each could be inhibited, offering multiple targets for entry inhibitors that should not exhibit cross-resistance. Two inhibitors interfering with the entry process have been approved for clinical use. Maraviroc prevents the interaction of gp120 with the co-receptor CCR5, and T20 (enfuvirtide, Fuzeon) binds to HR1 in gp41 and prevents the formation of the six-helix bundle (9–11).

**Figure 1.**
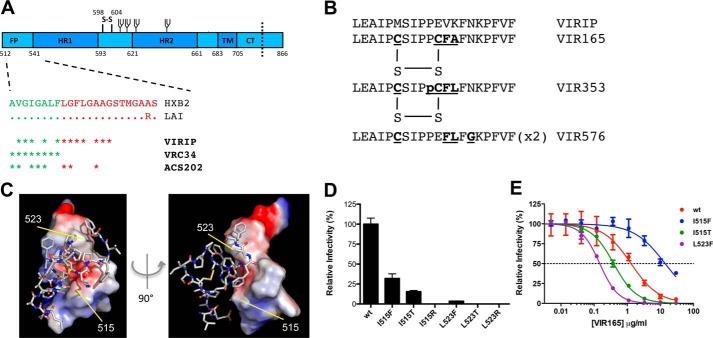
**Design of HIV-1_LAI_ fusion peptide mutations that cause resistance to VIR165.** *A,* schematic of the gp41 ectodomain. The various gp41 subdomains are indicated (*HR1* and *HR2,* heptad repeats 1 and 2; *MPER*, membrane proximal external region; *TM*, transmembrane domain; *CT*, cytoplasmic tail). The sequence of the HIV-1_LAI_ FP is aligned to the sequence of reference strain HIV-1_HXB2_. *Dots* indicate identical amino acids. FP residues accessible in the pre-CD4–bound state are depicted in *green*, nonaccessible residues in *red*. Contact residues within the FP with VIRIP, VRC34, and ACS202 are indicated with *asterisks* (14, 15). *B,* sequence of the natural peptide VIRIP and the more potent and stable derivative VIR165 and VIR353 cyclized by the introduction of a disulfide bond and the dipeptide VIR576. *p* indicates nonnatural amino acid d-proline. *C,* molecular model of VIR165 in complex with the HIV-1_LAI_ FP. VIR165 is shown in *sticks*, with the disulfide bond indicated in *yellow*. The FP is represented in a space-filling model colored based on the electrostatic surface potential (*red,* acidic; *blue*, basic), showing the large neutral hydrophobic area of the FP in *white*. The VIR165–FP model was drawn using PyMOL (DeLano Scientific; pymol.sourceforge.net) using PDB accession code 2JNR. Residues 515 and 523 of the FP are indicated. *D,* infectivity in single cycle infection experiments of virus variants containing substitutions in the FP at positions 515 or 523. Ile-515 and Leu-523 (I4 and L12 in gp41 numbering) were substituted to amino acids Thr, Arg, or Phe, to explore differences in amino acid sidechain size, charge, and hydrophobicity for their effect on the interaction with VIR165. *E,* inhibition of HIV-1_LAI_ variants containing the I515F, I515T, and L523F mutations by VIR165.

Münch *et al.* (12) identified another class of HIV-1–fusion inhibitors, termed anchor inhibitors, which supposedly target the FP (Fig. 1*A*). The prototype inhibitor VIRIP is a natural 20-amino acid breakdown product of α1-antitrypsin. A number of VIRIP-derivatives with enhanced stability and potency, such as VIR165, VIR353, and VIR576 have been described with various substitutions including two cysteines to create a disulfide bond (Fig. 1*B*). VIRIP and VIRIP-derivatives are thought to inhibit HIV-1 entry by interfering with FP function, which distinguishes VIRIP from classical fusion inhibitors that prevent six-helix bundle formation. The VIRIP-derivative VIR576 has been evaluated in a phase I trial of 10-day monotherapy in treatment naive individuals, resulting in a mean viral load reduction of 1.23 log_10_.

The ability of VIRIP to inhibit FP-mediated hemolytic activity and NMR analyses of the VIRIP–FP complex point at an inhibitory mechanism involving the FP (12, 13). The recent discovery that the FP can be targeted by broadly neutralizing antibodies, in particular VRC34 and ACS202, and that it might be a viable vaccine target, lends further support to the supposition that the FP is a viable drug target (14–17).

However, random mutagenesis studies could not identify FP substitutions that caused VIRIP resistance (12). Furthermore, *in vitro* escape studies with the VIRIP-derivative VIR353, which required unusually long-term virus culture (up to 90 passages), could not reveal mutations in the FP, but rather identified resistance mutations in the C4 (A433T) or C5 (V489I) domains of gp120 and the HR1 (L545M, V570I) or loop (A612T) domains of gp41 (18). Similar escape studies performed by our group also identified substitutions in the C1 domain of gp120 (V42I, A58V, A60E, E64K, and H66R) or the HR1 domain of gp41 (A558T and Q577R) (19). Interestingly a number of escape mutations in the C1 domain of the gp120 subunit (A60E, E64K, and H66R) rendered the virus dependent on the drug (19). These latter substitutions were found to stabilize the Env trimer and were useful in generating recombinant native-like (SOSIP) Env trimers (19, 20).

The absence of escape mutations in the FP created some controversy about the putative binding site of VIRIP and it was suggested that VIRIP may interact with an unidentified region of Env different from the FP (18, 21). Here we further unravel the mechanism of inhibition by VIRIP-like peptides. We show that designed mutations within the FP can alter the sensitivity of HIV-1 to VIR165. Furthermore, we show that VIRIP inhibits during an intermediate post-CD4–binding entry step that is overlapping but not identical to the step that is inhibited by HR2-based fusion inhibitors such as T20. Consistent with this we found that a subset of mutations that cause resistance against HR2-based fusion inhibitors can provide cross-resistance to VIR165, in particular those that are located outside the inhibitor-binding site and that might affect fusion kinetics. All these data are consistent with the idea that the FP is the actual drug target and that VIRIP and derivatives act during an early step in during entry that is overlapping but not identical to the formation of the six-helix bundle formation inhibited by traditional fusion inhibitors.

## Results

### Substitutions in the FP can alter sensitivity to VIR165

Most studies on the subject are consistent with the supposition that VIRIP analogs target the FP, but none of the evidence is direct (12, 19). To confirm that the FP is the target of the potent VIRIP-derivative VIR165, we designed a set of FP mutants (Fig. 1, *C* and *D*). We anticipated that this would not be straightforward because generally only hydrophobic residues are allowed in the FP to facilitate insertion into the target membrane. Indeed, hydrophilic residues are extremely rarely present in the FP of natural HIV-1 isolates (Los Alamos Database, www.hiv.lanl.gov/). Because VIR165 is thought to interact with the FP through hydrophobic interactions, conservative (hydrophobic) substitutions might not cause a significant level of resistance, whereas less conservative substitutions are expected to be deleterious to the FP function. This might explain why no FP escape mutants have been identified so far (12, 18).

Based on the NMR structure model of the VIR165–FP complex (Fig. 1*C*) (12), we identified two residues within FP that interact intimately with VIR165 through side chain interactions: Ile-515 and Leu-523. We substituted these relatively small amino acids for Thr, Arg, or Phe to explore differences in amino acid side chain size, charge, and hydrophobicity (22). Thr has only a minor impact on the amino acid size but represents a change from apolar to polar; Arg is also polar and increases the size of the side chain yet also has hydrophobic properties and can be accommodated in membranes as a “snorkeling Arg” (23); Phe maintains the hydrophobic character, but enhances the size of the side chain considerably.

We found that most substitutions at position 515 and 523 were deleterious for viral infectivity, but substitutions I515F and I515T and to a minor extend L523F were viable with relative infectivities ranging from 4 to 32% compared with WT (Table 1, Fig. 1*D*). When we tested the sensitivity to VIR165, we measured a 10-fold increase in resistance for I515F (Table 1, Fig. 1*D*), supporting the suggestion that the FP is the binding site of VIR165. This finding is consistent with the identification of the I515F polymorphism in an HIV-1–infected patient who responded poorly to VIR576 in a clinical trial (13). In contrast to I515F, the I515T and L523F substitutions rendered the virus more sensitive to VIR165, by 3- and 9-fold, respectively (Table 1, Fig. 1*D*). These substitutions might increase the affinity of the FP for VIR165. Thus, we show that FP substitutions can modulate the sensitivity to VIR165, strengthening the evidence that VIRIP analogs target the FP.

**Table 1:** **Relative infectivity and sensitivity to VIR165 of FP mutants I515F, I515T, and L523F**

HIV-1 LAI mutant	Relative infectivity	VIR165 IC_50_ (S.D.)	VIR165 IC_50_	Fold-resistance	Fold-sensitivity
	%	μ*g/ml*	*nm*		
WT	100	1.31 (0.23)	0.58		
I515F	32	14.05 (2.76)	6.27	**10.7**	
I515T	15	0.39 (0.02)	0.17	0.3	**3.3**
L523F	3	0.15 (0.01)	0.07	0.1	**8.8**

### VIR165 inhibits during an intermediate entry step

**Figure 2.**
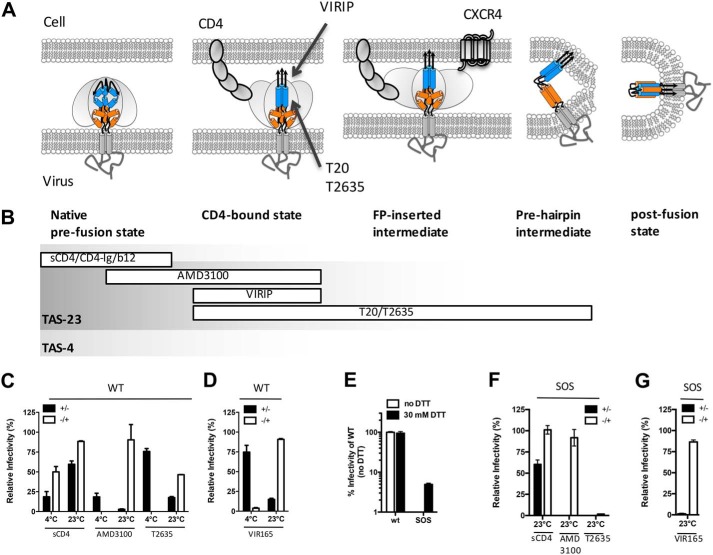
**VIR165 acts at an intermediate step of the HIV-1 entry process.** *A,* cartoons of different steps during HIV-1 entry. HIV-1 enters the cell by first binding to the CD4 receptor, which induces multiple conformational changes. Subsequently, binding to the CXCR4 or CCR5 co-receptor and insertion of the FP into the target cell membrane triggers formation of the stable six-helix bundle and fusion of the viral and cell membrane. *B,* entry and fusion inhibitors can act during different stages of the entry process. *C* and *D,* temperature-arrested state experiments: virus was allowed to attach to target cells at 4 or 23 °C in the presence or absence of inhibitor (temperature-arrested states at 4 and 23 °C; TAS-4 and TAS-23). Unbound virus and inhibitor were washed away after centrifugation and fresh medium was added that contained new inhibitor if the incubation during TAS-4 or TAS-23 was performed in the absence of inhibitor. *C,* inhibition of WT virus infection by sCD4, AMD3100, and T2635 during formation or post-TAS-4 and TAS-23. *Black bars* represent infectivity when the inhibitor was present during formation of TAS and *white bars* show the infectivity when the inhibitor was present post-TAS-4 or -TAS-23 formation. *D,* inhibition of WT virus when VIR165 was present during or post-TAS-4 or -TAS-23. *E–G,* SOS-arrested state experiments. *E,* infectivity of WT virus and SOS virus, which contains a disulfide bond between gp120 and gp41 allowing CD4 and co-receptor binding but blocking gp120 dissociation from gp41, as well as six-helix bundle formation. *White bars* represent infection without addition of DTT, whereas *black bars* represent infection after incubation for 10 min with 30 mm DTT. *F,* virus was allowed to attach to target cells at 23 °C in the presence (+/−) or absence of inhibitor (SOS-arrested state at 23 °C; SAS-23) before addition of 30 mm DTT for 10 min. Unbound virus, inhibitor, and DTT were washed away after this incubation and fresh medium was added that contained new inhibitor if the incubation during SAS-23 was performed in the absence of inhibitor (+/−). Inhibition of SOS virus infection by sCD4, AMD3100, and T2635 during formation or post-SAS-23. *Black bars* represent infectivity when the inhibitor was present during formation of SAS and *white bars* show the infectivity when the inhibitor was present post-SAS-23 formation. *G,* Inhibition of SOS virus when VIR165 was present during or post-SAS-23 formation.

To delineate which entry step was inhibited by VIR165, we performed temperature arrest experiments. Such experiments allow to distinguish between the initial receptor attachment events and the subsequent conformational changes that lead to membrane fusion (Fig. 2*A*) (24–28). In previous studies using these arrested state experiments no FP targeting agents were available. Here, we also compared and contrasted VIR165 with the third generation classical HR2-based fusion inhibitor T2635 (29).

First, WT HIV-1_LAI_ (WT) was allowed to bind target cells at either 4 or 23 °C for 2 h during spinocculation (30–32). At 4 °C the virus is able only to bind CD4 but further conformational changes needed for co-receptor binding do not occur. At 23 °C the virus will bind both CD4 and the co-receptor, but subsequent conformational changes leading to membrane fusion require a higher temperature of 37 °C (32–34). We refer to these temperature-arrested states as TAS-4 and TAS-23, respectively (Fig. 2*B*). Inhibitors can be added during formation of TAS-4 and TAS-23 or during the transition to 37 °C. One can thus assess whether compounds inhibit during the first step (*i.e.* approximately coincidental with CD4 binding), the second step (coincidental with CD4 binding or co-receptor binding), or the later steps (including fusion) of the entry process. We verified the assay by investigating at which stage WT virus was inhibited by a number of control reagents. Soluble CD4 (sCD4) targets the CD4-binding site on Env and only inhibited efficiently during formation of TAS-4 but not after, confirming that CD4 binding already takes place at 4 °C. In contrast, inhibition by the CXCR4 antagonist AMD3100 was more efficient after TAS-4 formation, indicating that co-receptor binding is not very efficient at 4 °C. However, AMD3100 inhibited virus infection only when added during formation of TAS-23 and not after, consistent with the notion that Env has already engaged the co-receptor during TAS-23, as described previously (33).

The classical fusion inhibitor T2635 (Fig. 2*C*) did not efficiently inhibit HIV-1 infectivity during formation of TAS-4, consistent with the notion that its target (HR1) is not yet exposed. When added after TAS-4 formation, T2635 was able to efficiently inhibit the entry process. In contrast, T2635 was able to inhibit virus infection when added during formation of TAS-23, but less so when added after TAS-23. Combined, these results indicate that HR1 exposure and pre-hairpin intermediate formation becomes accessible during the formation of TAS-23 and requires the shift from 4 to 23 °C. Binding of Env to the co-receptor coincides with becoming susceptible to fusion inhibitors. When T2635 was added after TAS-23, infection was partly inhibited indicating that HR1 remained exposed at least partially and/or temporarily. This is consistent with findings by Henderson and Hope (24) showing that the binding site of the fusion inhibitor C34 only becomes available during formation of TAS-23 but virus–cell fusion can proceed to a state preceding six-helix bundle formation and membrane fusion. Thus, exploiting TAS-4 and TAS-23, we are able to distinguish between the CD4 binding phase, the co-receptor binding phase, and the final stage of membrane fusion for which an increased temperature of 37 °C is necessary (34).

We next assessed at what stage VIR165 inhibited virus infection. Similar to the classical fusion inhibitor T2635, VIR165 was only able to inhibit infection when present post-TAS-4 and during formation of TAS-23 (Fig. 2*D*), indicating that VIR165 inhibits a similar intermediate entry step as T2635 after CD4 binding and before membrane fusion. This is consistent with the supposition that VIR165 inhibits fusion by binding to the FP, which should only be shortly exposed fully after CD4 binding and prior to insertion into the target membrane. On the other hand, whereas T2635 exhibited residual activity after TAS-23, the VIR165 peptide was completely inactive at this stage.

Next, we performed experiments in which a mutant virus was used with an engineered disulfide bond between gp120 and gp41, allowing CD4 and co-receptor binding, but blocking gp120 dissociation from gp41 and subsequent six-helix bundle formation (SOS-arrested state; SAS) (35, 36). SAS can be established at 37 °C, and consequently represents a later intermediate than TAS-23, in which hemifusion occurs immediately following SOS disulfide bond reduction (35, 36). Because we do not expect VIR165 to inhibit a very late entry step we performed the experiments at 23 °C and refer to the arrested state as SAS-23, which represents a doubly arrested intermediate restricted by suboptimal temperature and covalent linkage of gp120 and gp41.

HIV-1_LAI_ SOS virus (containing a disulfide bond between residues 501 in gp120 and 605 in gp41) was not infectious (37), but could be rendered infectious by adding 30 mm DTT after 2 h spinoculation at 23 °C for 10 min (Fig. 2*E*). AMD3100 inhibited SOS virus only when added before formation of SAS-23, but not after SAS-23 was formed, indicating that the co-receptor was already bound at SAS-23, consistent with the TAS-23 experiment (Fig. 2*F*)., T2635 was able to inhibit in both steps (Fig. 2*F*), confirming that HR1 becomes exposed prior to formation of SAS-23 or TAS-23, but remains partially and/or shortly exposed after reduction of the disulfide bond or incubation at 23 °C, indicating that the six-helix bundle does not form immediately. The virus was effectively inhibited when VIR165 was introduced prior to SAS-23 formation, but VIR165 was ineffective when added after SAS-23, indicating that the FP quickly became unavailable for binding (Fig. 2*G*), in contrast to the binding site of T2635. The efficient binding of VIR165 during SAS-23 suggests that it does not require the removal of gp120.

Thus, SAS and TAS experiments reveal that VIR165 inhibits at an intermediate, post-CD4–binding entry step that overlaps but is not identical to the step(s) inhibited by HR2-based fusion inhibitors T20 and T2635. This is consistent with the FP being fully exposed only shortly, after CD4-binding and before insertion into the target membrane. In contrast, the pre-hairpin intermediate that is the target of classical HR2-based fusion inhibitors such as T2635, is still temporarily exposed after FP insertion. However, inhibitors targeting the co-receptor–binding site would present with a similar phenotype in SAS and TAS assays. Therefore, these data alone are not sufficient to identify the FP as the target, but taken together with the mutagenesis data (Fig. 1) and the previously published data (12, 19), the FP appears the mostly likely target.

### Resistance mutations to HR2-based fusion inhibitors can provide cross-resistance to VIR165

Because VIR165 targets an intermediate Env state that is also susceptible to HR2-based fusion inhibitors, we tested whether HIV-1 variants that are resistant to three generations of fusion inhibitors, T20, T1249, or T2635, were cross-resistant against VIR165. Resistance to HR2-based fusion inhibitors can be caused by different mechanisms (22, 31, 39–43). First, mutations within the peptide-binding site can affect drug binding. Second, mutations in other gp41 domains can cause resistance, most likely by affecting the kinetics of the fusion process, *e.g.* by reducing the time window for the inhibitors to act (31, 39, 41–44). Because the window of opportunity for VIR165 and HR2-based fusion inhibitors partially overlap, some resistance mutations that affect this window by enhancing the fusion kinetics could possibly provide cross-resistance to VIR165. On the other hand, mutations in the fusion inhibitor-binding site would not be expected to do so because the VIR165-binding site is different.

We tested a number of substitutions that were previously identified as resistance mutations for fusion inhibitors. HR1 substitution V549A is a common T20-resistance mutation (45, 46). In fact, Val-549 located in the binding site of HR2-based inhibitors, is a resistance “hot spot” for different generations of HR2-based peptidic fusion inhibitors (45, 47–49). Furthermore, the HR1–HR2 double mutant V549A/N637K causes T20 dependence (44). Substitutions located at the edge (Q577R) or outside the binding site of HR2-based fusion inhibitors (Q590E, K601E, and N637K) provide resistance to the more potent second and third generation peptides T1249 and T2635 (40, 47). Several combination mutants with three to five substitutions in gp41 that were identified in T2635-escape studies were also included (40).

We performed single cycle infection assays using the TZM-bl reporter cell line to determine the IC_50_ of VIR165 against these variants (Table 2). The classical T20-resistance mutation V549A did not confer resistance, neither did the combination of V549A and N637K that caused T20-dependence (44, 50). However, the Q577R variant showed considerable resistance to VIR165 (>7.5-fold; Table 2). Of note is that this substitution was also selected in a previous VIR165 escape study (19). All combination mutants containing the Q577R substitution were also resistant to VIR165, although the additional substitutions did not raise the level of resistance, suggesting that the Q577R substitution was the major contributor to VIR165 resistance. The Q590E and K601E substitutions provided low-level or moderate VIR165 resistance (2.8- and 3.4-fold, respectively). Thus, resistance mutations in the gp41 loop domain or C-terminal part of HR1 can confer cross-resistance to VIR165, whereas those in the middle of HR1 (V549A) or HR2 (N637K) cannot, supporting the supposition that VIR165 and classical fusion inhibitors target an overlapping but nonidentical step in virus entry. HR2 mutations are proposed to alter the kinetics of HR1–HR2 association and six-helix bundle formation, thereby decreasing the time available for HR2-based fusion inhibitors (39, 42, 50). The fact that this process does not affect VIR165 inhibition is consistent with the idea that the FP is already inserted into the target membrane before six-helix bundle formation. Likewise, altered kinetics of six-helix bundle formation do not affect sensitivity to FP targeting inhibitors. This suggests that HR2-based fusion inhibitors act during an overlapping but extended time window compared with VIR165.

**Table 2:** **VIR165 cross-resistance to HIV-1 mutants resistant to different generations of HR2-based fusion inhibitors**

HIV-1 LAI mutant	Phenotype	Ref.	VIR165 sensitivity			Env stability
			IC_50_ (S.D.)	IC_50_	Fold-resistance	(*T_m_*)
			μ*g/ml*	*nm*		
WT	Sensitive		3.9 (0.34)	1.74	1.1	43.6
V549A	T20-resistant	22	3.4 (0.76)	1.52	1.0	44.1
Q577R	T2635-resistant	40	>30	>13	**>7.5**	43.4
K588E	T20/T2635-resistant*^a^*	40	3.1 (0.46)	1.38	0.8	40.8
K588N	T20-resistant	40	4.9 (0.33)	2.19	1.3	41.9
Q590E	T1249/T2635-resistant	40, 47	9.9 (0.81)	4.42	**2.8**	41.1
K601E	T20/T1249/T2635-resistant	40, 47	13.4 (1.77)	5.98	**3.4**	40.8
T605N	T2635-resistant	40	5.8 (0.49)	2.59	1.5	43.3
N611D	Sensitive	40	8.6 (0.75)	3.84	**2.2**	43.1
N624D	Sensitive	40	5.6 (0.29)	2.50	1.5	42.8
N637K	T20/T2635-resistant*^a^*	40	4.6 (0.74)	2.05	1.2	43.0
A517V	FP mutant	40	4.1 (0.30)	1.83	1.1	43.8
V549A/N637K	T20-dependent	44	2.9 (0.33)	1.29	0.7	
Q590E/N636S/N637K	T1249/T2635-resistant	40, 47	8.5 (1.0)	3.03	**2.2**	41.3
Q577R/N624D	T2635-resistant	40	>30	>13	**>7.5**	41.9
K588E/N637K/E647G (H2A)	T2635-resistant*^b^*	40	3.4 (0.27)	1.52	1.0	41.2
A517V/L544S/Q577R/N637K/H643Q/E647G (H3C)	T20/T2635-resistant*^a^*	40	>30	>14	**>7.5**	40.8
L544S/K588N/T605N/N637K/E662G (H5C)	T20/T2635-resistant*^a^*	40	3.3 (0.35)	1.47	0.9	40.6
L544S/Q577R/N624D/N636S/N637K/H643Q (H29C)	T20/T2635-resistant*^a^*	40	>30	>13	**>7.5**	41.7
Q577R/N611D/N636S/N637K/H643Q/K665Q (H28C)	T20/T2635-resistant*^a^*	40	>30	>13	**>7.5**	42.5

*^a^* No T1249 resistance data available.

*^b^* No T20 and T1249 resistance data available.

### Residues 588, 590, and 601 affect Env spike stability

Residues 577, 590, and 601 that provide resistance to first and third generation HR2-based inhibitors T20 and/or T2635 (47) also cause cross-resistance to VIR165, suggesting that they might accelerate the step in which FP and HR1 are simultaneously exposed, *i.e.* the intermediate prior to FP insertion (Fig. 2*A*, *middle panel*). Resistance to fusion inhibitors such as T20 and T2635 generally correlates with Env stability (43, 44, 50, 52–54). Env proteins with enhanced fusion kinetics and reduced HR1 exposure are often less stable compared because such Env proteins are more prone to undergo conformational changes down the fusion pathway (52, 53).

**Figure 3.**
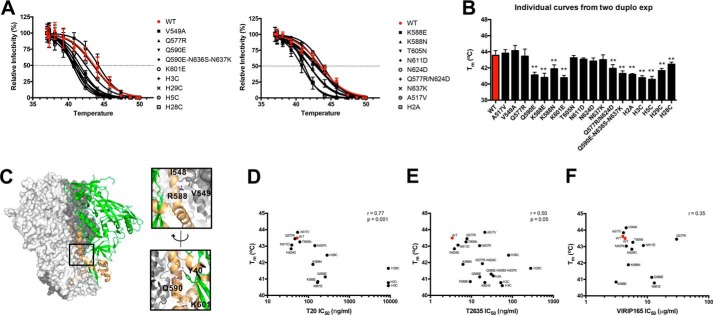
**The effect of Env stability on resistance to fusion inhibitors.** *A,* the thermostability of WT and mutant viruses was determined by incubating them for 1 h at escalating temperatures, followed by testing the remaining infectivity on TZM-bl reporter cells. A representative curve of the residual infectivity after incubation is plotted for each individual mutant virus. For viruses containing one, two, or three substitutions these substitutions are listed. Viruses containing more than 3 mutations are abbreviated as follows: H2A (K588E/N637K/E647G); H3C (A517V/L544S/Q577R/N637K/H643Q/E647G); H5C (L544S/K588N/T605N/N637K/E662G); H28C (Q577R/N611D/N636S/N637K/H643Q/K665Q); H29C (L544S/Q577R/N624D/N636S/N637K/H643Q). See also Table 2. These viruses were obtained from escape studies with T2635 as described previously (26). *B,* the mean midpoints of thermal denaturation (*T_m_*) and their mean ± S.E. of WT and mutant viruses are shown, measured in two independent experiments, each performed in duplicate. Significant differences compared with WT are indicated with: **, *p* < 0.005. The raw data of one such experiment are depicted in *A*. The exact values are also listed in Table 2. *C,* location and contacts of residues 588, 590, and 601. The Env trimer structure (PDB 5CEZ) is shown with one protomer shown (gp120, *green*; gp41, *pale orange*). Structure was drawn using PyMOL (DeLano Scientific). The *two insets* on the *right* reveal the interactions of residues 588 (*upper right panel*), and 590 and 601 (*lower right panel*). Table 3 lists the contact residues for each residue (*middle column,* distance of <4 Å between atoms of two residues; *right panel*, distance of <5 Å). The most evident contact residues are depicted in the structural model and are listed in *bold. D–F,* correlation plots showing the relationships between Env stability of mutant viruses and resistance to fusion inhibitors T20 (*D*), T2635 (*E*), and VIR165 (*F*). The *r* and *p* values for nonparametric Spearman correlations are shown.

We measured the thermal stability of a series of viruses containing one or more amino acid substitutions associated with resistance to HR2-based fusion inhibitors, including Q577R, Q590E, and K601E viruses that cause VIR165 cross-resistance. This panel of viruses covers a large range in resistance levels to the fusion inhibitors T20 and T2635 (40, 44, 47), and cross-resistance to VIR165 (Table 2, Fig. 3). Each virus was incubated for 1 h at increasing temperatures and the residual infectivity was measured using TZM-bl reporter cells to determine the relative remaining Env function (Fig. 3, *A* and *B*).

The WT virus had a midpoint of thermal denaturation (*T_m_*) of 43.6 °C (Fig. 3*A*, Table 2), which is in the same range as those reported for multiple different viruses including HIV-1_LAI_ (55, 56).^4^ The mutant viruses had *T_m_* values ranging from 40.6 to 44.1 °C (Fig. 3, *A* and *B,* Table 2). The K588E, Q590E, and K601E mutant viruses showed the most substantial decrease in thermal stability compared with WT virus, with *T_m_* values of 40.8, 41.1, and 40.8 °C, respectively, *versus* 43.6 °C for WT virus (Fig. 3*B*). In contrast, a Q577R substitution did not affect thermal stability (*T_m_* value 43.3 °C *versus* 43.6 °C for WT virus). Inspection of the local structure might explain these effects. Residue 588 is in close proximity to Ile-548 and Val-549 mediating long-distance contacts within gp41, whereas residues 590 and 601 are in contact with residue 40 in the C1 domain of gp120 and influence inter-subunit interactions (Fig. 3*C*, Table 3).

**Table 3:** **Contact residues of Env positions 588, 590, and 601**

Residue	Contact residues <4 Å*^a^*	Contact residues <5 Å
Arg-588	584–592 (same protomer)	584–592 (same protomer)
	Leu-545, Ile-548, Val-549 (other protomer)	Leu-545, Ile-548, Val-549 (other protomer)
Gln-590	Tyr-40, 586–594	Tyr-40, 586–594, 599
Lys-601	593, 598–604	Tyr-40, 593, 597–604

*^a^* Distance calculations based on molecular structure PDB 5CEZ (38) using PyMol (DeLano Scientific).

Overall, there was a very strong correlation between Env stability and resistance to T20 (Fig. 3*D*) and a moderate correlation with resistance to T2635 (Fig. 3*E*), supporting the link between Env stability and resistance to HR2-based fusion inhibitors. However, there was no such relationship for VIR165 resistance (Fig. 3*F*). Thus, whereas the Q590E and K601E substitutions might provide resistance to HR2-based fusion inhibitors and VIR165 by destabilizing the pre-fusion conformation of Env, such a relationship was not a general one for VIR165. These data are consistent with nonidentical mechanisms of action and/or windows of opportunity for fusion and anchor inhibitors.

## Discussion

Münch *et al.* (12) showed that VIRIP and derived peptide variants inhibit HIV-1 entry, likely by targeting the FP. Various lines of evidence suggested that VIRIP inhibits by binding to the FP and thus blocking its insertion into the target cell membrane. However, random mutagenesis studies and virus escape studies did not reveal any resistance mutations within this putative binding site, but rather identified escape mutations in the C4 and C5 domains in gp120 and the HR1 domain in gp41 (12, 18, 19), shedding doubt about whether or not the FP is the target of VIRIP derivatives.

Here we further explored the mode of action of VIR165, one of the most potent VIRIP-derivatives. We show that VIR165 inhibits an intermediate post-CD4–binding step that is overlapping with, but not identical to the step inhibited by HR2-based fusion inhibitors. VIR165 likely inhibits a form of the CD4-triggered pre-hairpin intermediate that has not yet inserted its FP into the target membrane, whereas HR2-based inhibitors like T20 are also able to inhibit the pre-hairpin intermediate after insertion of the FP into the membrane (Fig. 2*A*).

Interestingly, the FP targeting bNAbs ACS202 and VRC34 are able to bind to the FP prior to CD4 engagement, in contrast to VIR165, which is only able to bind post-CD4 binding. A likely explanation of this paradox can be found in the required contact residues within the FP (Fig. 1*A*). VRC34 and ACS202 interact with the N terminus of gp41, in particular with the first eight residues (512–519) (Fig. 1*A*) (14, 15). In contrast, VIRIP also interacts intimately with residues 519–527 (12). Based on Env trimer structures (15) only the N-terminal part of the FP is accessible in the pre-CD4–bound state, including most or all residues required for ACS202 and VRC34 binding, but not all critical contact residues of VIRIP and its derivatives.

The most common drug-resistance mechanism is decreased drug-target affinity by substitutions in the drug-binding site (57) and this has been described repeatedly for HIV-fusion inhibitors (22). Although no studies have described virus escape mutations in the FP for VIRIP-derivative peptides (12, 18), we were able to design FP substitutions that altered sensitivity to VIR165 (Fig. 1, *C–F*). Interestingly one of these substitutions was also present in an infected patient that responded poorly to VIR576 (13). The lack of spontaneous escape mutations in FP might be explained by the conserved and hydrophobic nature of this domain (58–64). VIRIP–FP interactions are almost exclusively mediated by hydrophobic interactions (Fig. 1*A*). Such hydrophobic interactions can be rather promiscuous or polyspecific, a property referred to as “hydrophobic stickiness” (65–70), which may explain why no resistance mutations occur in FP as the only allowed (hydrophobic) substitutions in FP may not significantly perturb VIR165 binding. Thus, HIV-1 might not be able to escape from VIRIP-derivatives via mutation of the FP because the penalty on Env function and viral fitness is too high. In addition, the Ile to Phe substitution at position 515 in the FP, which increases VIR165 resistance by 10-fold, requires a difficult codon change with two point mutations, representing a higher genetic barrier for virus evolution than substitutions caused by a single point mutation in other Env domains that were apparently selected for VIRIP resistance (18, 19).

Env stability is strongly correlated with resistance to the HR2-based fusion inhibitor T20 and moderately with resistance to T2635 (Fig. 3, *D* and *E*). However, this correlation does not translate to resistance to VIR165 (Fig. 3*F*), except for the specific cases of substitutions Q590E and K601E, previously identified in a fusion inhibitor escape study (47). These substitutions are associated with a decreased thermal stability of Env (Fig. 3, *A* and *B*). The destabilization of the pre-fusion conformation of Env probably alters the fusion kinetics and results in the resistance to HR2-based fusion inhibitors, as well as VIR165. However, we could not identify a general link between Env stability and resistance against VIR165.

Taken together our data support the hypothesis that VIRIP derivatives inhibit an intermediate step during virus entry and target the FP. Furthermore, we show that these peptides inhibit a step that is nonidentical but overlapping with the step inhibited by classical HR2-based fusion inhibitors.

## Experimental procedures

### Reagents

MAbs and inhibitors were obtained as gifts, or purchased, from the following sources: William Olson (Progenics Pharmaceuticals) provided soluble CD4 (sCD4). AMD3100 was a generous gift from Dr. D. Schols (Rega Institute, Leuven University).

### Peptide synthesis

Peptides VIR165 (LEAIPCSIPPCFAFNKPFVF), T20 (YTSLIHSLIEESQNQQEKNEQELLELDKWASLWNWF), and T2635 (TTWEAWDRAIAEYAARIEALIRAAQEQQEKNEAALREL) were synthesized as described previously (22, 47). The peptides were lyophilized from acetonitrile (50% (v/v) in water) and stored at −20 °C.

### Infectivity and IC_50_ determination

The TZM-bl reporter cell line (71, 72) stably expresses high levels of CD4 and HIV-1 co-receptors CCR5 and CXCR4 and contains the luciferase and β-galactosidase genes under the control of the HIV-1 long-terminal repeat promoter. The TZM-bl cell line was obtained through the National Institutes of Health AIDS Research and Reference Reagent Program, Division of AIDS, NIAID, National Institutes of Health (John C. Kappes, Xiaoyun Wu, and Tranzyme Inc. (Durham, NC)). One day prior to infection, 17 × 10^3^ TZM-bl cells per well were plated on a 96-well plate in Dulbecco's modified Eagle's medium containing 10% fetal bovine serum and penicillin-streptomycin (both at 100 units/ml) and incubated at 37 °C with 5% CO_2_. A fixed amount of virus (1.0–5.0 ng of CA-p24) was preincubated for 30 min at room temperature with serial dilutions of VIR165. This mixture was added to the cells in the presence of 400 nm saquinavir (Roche, Mannheim, Germany) to block secondary rounds of infection and 40 μg/ml of DEAE in a total volume of 200 μl. DEAE was added to enhance HIV-1 infectivity without affecting the sensitivity to inhibitors tested. Two days post-infection, the medium was removed and cells were washed once with PBS (50 mm sodium phosphate, pH 7.0, 150 mm NaCl; PBS) and lysed in reporter lysis buffer (Promega, Madison, WI). Luciferase activity was measured using a luciferase assay kit (Promega, Madison, WI) and a Glomax luminometer according to the manufacturer's instructions (Turner BioSystems, Sunnyvale, CA). All infections were performed in duplicate in at least two independent experiments. Uninfected cells were used to correct for background luciferase activity. The infectivity of each mutant without inhibitor was set at 100%. Nonlinear regression curves were determined and 50% inhibitory concentrations (IC_50_) were calculated using Prism software version 5.0.

### Temperature-arrested state-inhibition assays (TAS)

Temperature-trap experiments were performed using the spinoculation technique (30, 31). TZM-bl cells were plated as described above and were cooled to 4 °C before 5.0 ng of CA-p24 of cold virus was added. High concentrations (adjusted according to the potency for each inhibitor) of sCD4 (10 μg/ml), AMD3100 (10 ng/ml), 2F5 (10 μg/ml), T20 (300 ng/ml), T2635 (100 ng/ml), or VIR165 (10 μg/ml) were added when applicable and plates were centrifuged at 4 or 23 °C at 1,100 × *g* for 2 h. The cells were washed with cold or room temperature PBS to remove unbound virus and inhibitor. Fresh medium containing high concentrations of inhibitor was added when applicable and cells were incubated at 37 °C to allow complete membrane fusion and virus entry. Virus infectivity was measured in the presence of 400 nm saquinavir and 40 μg/ml of DEAE as described above. Cells were lysed 2 days post-infection and luciferase activity was measured to quantify virus infectivity. Infectivity of virus without inhibitor present at any of the steps was set at 100% for each temperature incubation.

### SOS-arrested state-inhibition assays

HIV-1_LAI_ SOS virus (containing a disulfide bond between residues 501 in gp120 and 605 in gp41) was not infectious (35–37), but could be rendered infectious by adding 30 mm DTT at 23 °C for 10 min. SOS arrest experiments were performed as the temperature-arrested state experiments with the addition of an extra 10-min incubation at room temperature with 30 mm DTT after the 2-h spinocculation at 4 or 23 °C. DTT was washed away before addition of medium containing the indicated inhibitors.

### Construction of HIV-1_LAI_ molecular clones

The full-length molecular clone of HIV-1_LAI_ (pLAI) (73) was used to produce WT and mutant viruses. The plasmid pRS1 was used to introduce mutations as described previously (47, 74) and the entire *env* gene was verified by DNA sequencing. Mutant *env* genes in pRS1 were cloned back into pLAI as SalI–BamHI fragments. Each virus variant was transiently transfected in C33A or 293T cells by calcium phosphate precipitation as previously described (75) or Lipofectamine 2000 according to the manufacturer's protocols (Invitrogen). The virus containing supernatant was harvested 3 days post-transfection and stored at −80 °C and the virus concentration was quantitated by capsid CA-p24 ELISA as described previously (51).

### Virus thermostability experiments

WT and mutant viruses were incubated for 1 h at a temperature range of 37 to 50 °C using a thermocycler before testing their residual infectivity on TZM-bl reporter cells as above. The midpoint of thermal denaturation (*T_m_*) was defined as the temperature at which 50% residual infectivity was observed. Average values are shown of at least two independent experiments performed in duplicate.

## Author contributions

D. E., J. P. L., B. B., and R. W. S. conceptualization; D. E., I. B., and S. W. d. T. data curation; D. E., I. B., S. W. d. T., and R. W. S. formal analysis; D. E., I. B., S. W. d. T., J. P. L., and R. W. S. investigation; D. E., I. B., S. W. d. T., and R. W. S. methodology; D. E., I. B., S. W. d. T., B. B., and R. W. S. writing-original draft; D. E., I. B., S. W. d. T., and R. W. S. writing-review and editing; J. P. L., B. B., and R. W. S. supervision; B. B. and R. W. S. funding acquisition.

## Footnotes

4 D. Leaman and M. Zwick, personal communication.

## Footnotes

3 The abbreviations used are:

Env: envelope glycoprotein
sCD4: soluble CD4
gp: glycoprotein
FP: fusion peptide
SAS: SOS-arrested state
TAS: temperature-arrested state.
