# Correction: HIV-1 anchor inhibitors and membrane fusion inhibitors target distinct but overlapping steps in virus entry

**DOI:** 10.1016/j.jbc.2022.101811

**Published:** 2022-03-12

**Authors:** Dirk Eggink, Ilja Bontjer, Steven W. de Taeye, Johannes P.M. Langedijk, Ben Berkhout, Rogier W. Sanders

Funding and additional information should read as follows:

This work was supported by 10.13039/100007553Aids Fonds Netherlands Grants 2005021 and 2008013, the 10.13039/100000865Bill and Melinda Gates Foundation Collaboration for AIDS Vaccine Discovery (CAVD) Grants OPP1111923 and OPP1132237, 10.13039/100000002National Institutes of Health Grant P01 AI110657 and funding from 10.13039/100010661European Union’s Horizon 2020 research and innovation programme under grant agreement No 681137.

